# Mevalonate inhibits acid sphingomyelinase activity, increases sphingomyelin levels and inhibits cell proliferation of HepG2 and Caco-2 cells

**DOI:** 10.1186/s12944-015-0137-8

**Published:** 2015-10-22

**Authors:** Ying Chen, Shu-Chang Xu, Rui-Dong Duan

**Affiliations:** Gastroenterology and Nutrition Laboratory, Department of Clinical Sciences, University of Lund, S 22184 Lund, Sweden; Gastroenterology, Tongji Hospital, Tongji University Medical School, 200065 Shanghai, China

**Keywords:** Mevalonate, Sphingomyelin, Cholesterol, Acid sphingomyelinase, Cell proliferation, Phosphatidylcholine, HepG2, Caco-2

## Abstract

**Background:**

Sphingomyelin (SM) and cholesterol are two types of lipid closely related biophysically. Treating the cells with exogenous sphingomyelinase (SMase) induces trafficking of cholesterol from membrane to intracellular pools and inhibition of cholesterol synthesis. In the present work, we address a question whether increased cholesterol synthesis affects hydrolysis of SM by endogenous SMases.

**Methods:**

Both HepG2 and Caco-2 cells were incubated with mevalonate. The SMase activity was determined and its mRNA examined by qPCR. The cellular levels of cholesterol, SM, and phosphatidylcholine (PC) were determined and cell proliferation rate assayed.

**Results:**

We found that mevalonate dose-dependently decreased acid but not neutral SMase activity in both HepG2 and Caco-2 cells with HepG2 cells being more sensitive to mevalonate. Kinetic examination in HepG2 cells revealed that acid SMase activity was increasing with cell proliferation, and such an increase was reversed by mevalonate treatment. Acid SMase mRNA was not significantly decreased and Western blot showed signs of proteolysis of acid SMase by mevalonate. After mevalonate treatment, the levels of cholesterol were significantly increased associated with increases in SM and PC. The cell growth was retarded by mevalonate and the effect was more obvious in HepG2 cells than in Caco-2 cells.

**Conclusion:**

Mevalonate can trigger a mechanism to enhance SM levels by inhibition of acid SMase. The effect may ensure the coordinate changes of SM and cholesterol in the cells. Mevalonate also affects cell growth with mechanism required further characterization.

## Introduction

Cholesterol and sphingomyelin (SM) are two important lipid constituents of mammalian cell membranes and are also co-localized in lysosomes, endosomes, intestinal vesicles, plasma lipoproteins, and fat milk globular membrane. The coexisting is due to the strong biophysical interactions between the polar group of SM with the hydroxyl group of cholesterol [1, 2]. Such an interaction affects the integrity and permeability of the cell membrane and has been considered to be important for survival of the cells. Although the existence of lipid rafts in plasma membrane where the cholesterol, sphingolipids and protein receptors are tightly packed [3–5] has been recently challenged by studies employed high-resolution secondary ion mass spectrometry [6, 7], the fact that interaction of cholesterol and sphingolipids affects cellular signaling is essentially not altered. Hydrolysis of SM has been shown to generate both antiproliferative molecules such as ceramide and sphingosine, and proliferative molecules such as sphingosine-1-phosphate (S1P) and ceramide-1-phosphate (C1P) [8, 9]. Cholesterol can also interact with membrane proteins and scaffold proteins affecting signal transduction pathways [7]. In addition, abnormal metabolism of both cholesterol and SM in plasma may play important roles in pathogenesis of atherosclerosis [10]. It is therefore important to understand the regulation of the homeostasis between these two types of lipid.

Cholesterol and SM can be synthesized in mammalian cells. The rate-limiting enzyme for cholesterol synthesis is 3-hydroxy-3-methyl-glutaryl-CoA reductase (HMGR), which catalyzes the formation of mevalonate from HMG-CoA. The mevalonate formed will undergo a mevalonate pathway resulting in formation of cholesterol [11]. Mevalonate pathway also generates non-sterol products which can affect gene translation and cell proliferation *via* mechanism related to post-transcriptional regulation [11, 12]. The SM levels are affected by both de novo biosynthesis and degradation. The serine palmitoyltransferase (SPT) is the rate-limiting enzyme that triggers the de novo SM synthesis. SM is degraded mainly by acid and neutral SMases, two types of ubiquitous enzymes in mammalian cells. Acid SMase (SMPD1) is a lysosomal enzyme that degrades SM internalized or transported in the intracellular vesicles [13], thus playing important roles in regulating cellular SM levels. It can also be transported to the membrane to hydrolyze membrane bound SM and trigger signal transduction pathways [14]. Neutral SMase has multiple forms including nSMase 1 (SMPD2), nSMase 2 (SMPD3), nSMase 3 (SMPD4) and MA-nSMase (SMPD5) with different biochemical properties and cellular locations [15]. The best studied neutral SMase is nSMase 2 that plays important roles in SM metabolism, cell signaling, tumorigenesis and bone homeostasis. The newly identified MA-nSMase may be involved in mitochondria related apoptosis [15]. Liver is an active organ that synthesizes both cholesterol and SM and secrets both products into circulation and gut. Liver is also an organ with higher acid SMase activity compared with many other organs such as intestine and pancreas [16, 17]. Comparing with cholesterol, diet has less influence on SM levels in plasma, because digestion of SM in the gut is incomplete, and sphingosine, the hydrolytic product from SM, is largely not resynthesized to SM in enterocytes and not entering the circulation in a decent amount [18, 19].

Because cholesterol and SM are two major lipid constituents in cell membrane, cells must have dedicate mechanisms to regulate the homeostasis of SM and cholesterol. Previous studies revealed that the levels of cholesterol in plasma membrane are affected by the membrane levels of SM. Treating fibroblasts with exogenous SMase caused a rapid translocation of cholesterol from membrane to intracellular pools [20–22]. Not only on the trafficking of cholesterol, hydrolysis of membrane SM also inhibits synthesis of cholesterol by inhibiting the activity of HMGR [23]. While previous studies focused mainly on the effect of changed SM levels on cholesterol homeostasis, fewer studies pay attention on the potential influence of cellular cholesterol on enzymes that hydrolyze SM. The present study addresses a question whether increased cholesterol synthesis induced by providing exogenous mevalonate can affect SM and SM hydrolytic enzymes in HepG2 liver cells and Caco-2 intestinal cells.

## Results

### Mevalonate reduces acid SMase activity in HepG2 and Caco2 cells

**Fig. 1:**
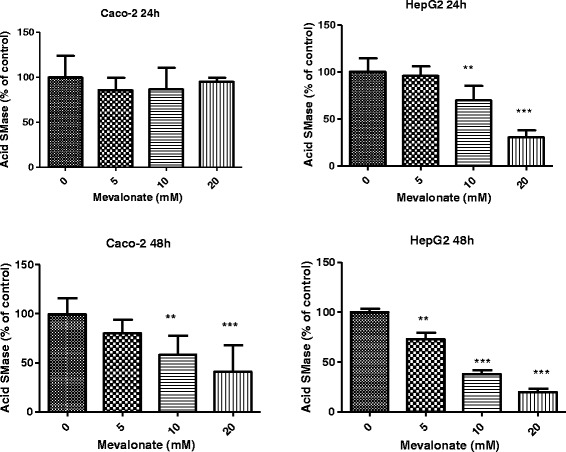
Dose dependent effects of mevalonate on acid SMase activity in HepG2 and Caco2 cells. The cells were incubated with mevalonate at different concentrations for 24 and 48 h. The acid SMase activity in the cell-free extracts was determined using choline labeled ^14^C-SM as substrate. The activity was adjusted to protein levels in the sample and expressed as percentage of the control (0 concentration of mevalonate). Results are means ± SD obtained from triple culture wells in three separate experiments. **p < 0.01, ***p < 0.005 compared with the control

After incubating HepG2 and Caco-2 cells with mevalonate, we found that the activities of acid SMase were decreased (Fig. 1). After 24 h incubation, mevalonate significantly inhibited acid SMase activity in HepG2 cells in a dose dependent manner. The inhibitory effect was enhanced after 48 h incubation, and at this time point 5 mM mevalonate, which did not show positive effect at 24 h incubation, became effective. Caco-2 cells appeared less sensitive than HepG2 cells to mevalonate treatment, because 24 h incubation with mevalonate at the same concentrations did not show similar effects and longer time incubation was required for reducing acid SMase activity in this type of cells. Neutral SMase activities were low and no significant changes were identified for both cell lines under the same conditions as above (Table 1).

**Table 1:** Changes of neutral SMase activity (pmol/h/mg protein) after mevalonate treatment in Caco-2 and HepG2 cells

Cell line	Time (h)	Mevalonate (mM)			
		0	5	10	20
Caco-2	24	100 ± 11.9	103.1 ± 26.5	107.3 ± 10.0	97.1 ± 30.7
	48	100 ± 14.9	108.0 ± 7.9	88.5 ± 17.8	92.7 ± 12.5
HepG2	24	122.4 ± 16.5	135.1 ± 23.8	118.9 ± 13.2	146.3 ± 39.7
	48	129.8 ± 8.5	129.6 ± 11.4	121.5 ± 7.9	125.1 ± 10.4

The cells were treated with mevalonate for 24 and 48 h. The cell lysates were prepared and overall neutral SMase activities in the supernatant were determined. Results are expressed as means ± SD obtained from triple samples for each concentration in separate three experiments

### Kinetic observation of the effect of mevalonate on acid SMase

**Fig. 2:**
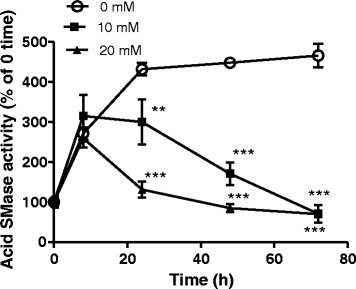
Kinetic changes of acid SMase in HepG2 cells in the absence and presence of mevalonate. The cells were subcultured in different wells for different time point examinations. One day after subculture, mevalonates at 0, 10 and 20 mM were added to the wells followed by incubation for 8, 24, 48 and 72 h. The acid SMase activities were determined at different time points. Results are means ± SD from triple wells for each time points in separated three experiments. **p < 0.01, ***p < 0.005 compared with 0 concentration

To examine the kinetic changes of acid SMase activity in the presence and absence of mevalonate, HepG2 cells were incubated with 10 and 20 mM of mevalonate for 8, 24, 48 and 72 hours. The changes of acid SMase were followed at different time points. The results are shown in Fig. 2. As can be seen, the acid SMase activity in nontreated group increased rapidly with the time up to 24 h when the cells were under rapid proliferation. Thereafter, the increase in acid SMase was leveled off and remained at high levels up to 72 hours. However, in the presence of mevalonate, the increase tendency of acid SMase activity was blocked after 8 h and reversed thereafter. Mevalonate at 20 mM was more effective than that at 10 mM. After 72 h incubation, mevalonate at both doses suppressed acid SMase activity to the level almost equal to that at the beginning (0 time).

### Changes of acid SMase mRNA and protein after mevalonate treatment

**Fig. 3:**
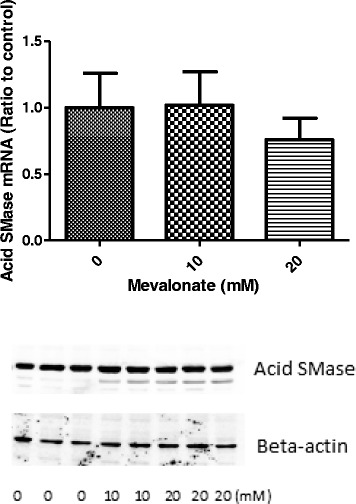
Changes of acid SMase mRNA (upper panel) and Western blot for acid SMase protein (lower panel) in HepG2 cells. The cells were incubated with mevalonate at different concentrations for 24 h. The total RNA was extracted and cDNA synthesized. QPCR was performed on Bio-Rad iCycler system. The results were normalized with the levels of the housekeeping gene (GAPDH) and expressed as ratio of the control. The data are expressed as means ± SD obtained from duplicate culture wells in three separate experiments. The Western blot (lower panel) for acid SMase was performed in HepG2 cells incubated with mevalonate at concentrations indicated for 24 h. No statistical significance was identified

The changes of acid SMase mRNA in HepG2 cells after 24 h incubation was determined by real time PCR. Although both 10 mM and 20 mM mevalonate inhibited acid SMase activity, reduction of acid SMase mRNA, about 20 %, was only found in the cells treated with 20 mM mevalonate (Fig. 3). However the statistical significance was not reached and the P value was 0.061 as compared with the non-treated cells. Western blot for acid SMase did not show significant changes of the enzyme protein induced by either 10 or 20 mM mevalonate (Fig. 3). However, in all mevalonate treated samples there was a faint band below the acid SMase band, which might be a proteolytic product derived from acid SMase.

### Increased contents of cholesterol, sphingomyelin and phosphatidylcholine by mevalonate

**Fig. 4:**
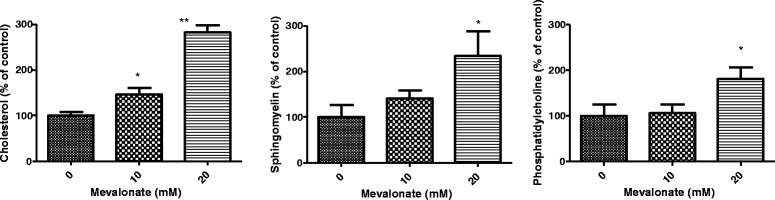
Quantification of SM, PC and cholesterol in the cells after mevalonate treatment. For SM and PC assay, HepG2 cells were first incubated with ^3^H-choline chloride to label both SM and PC for 48 h. After washing out the excess ^3^H-choline chloride, the cells were incubated with mevalonate for 24 h. The lipids were extracted and analyzed by TLC. The SM and PC bands were scraped according to the standards and the radioactivity measured by liquid scintillation. The cholesterol was measured by a commercial kit from Cayman. Results are means ± SD from duplicate culture wells in three separated experiments. * < 0.05, **p < 0.01 compared with nontreated cells

To evaluate whether mevalonate changes the metabolism of cholesterol and SM, the contents of cholesterol, SM and PC in HepG2 cells were determined. As shown in Fig. 4, after 24 h incubation, 10 and 20 mM mevalonate increased cellular cholesterol levels by about 46 % and 175 %, respectively (left panel). Increase in SM contents (middle panel) was also demonstrated but statistical significance was only found for 20 mM mevalonate treatment. Mevalonate at this concentration also significantly increased the PC levels in the cells.

### Mevalonate inhibits cell growth in both HepG2 and Caco-2 cells

**Fig. 5:**
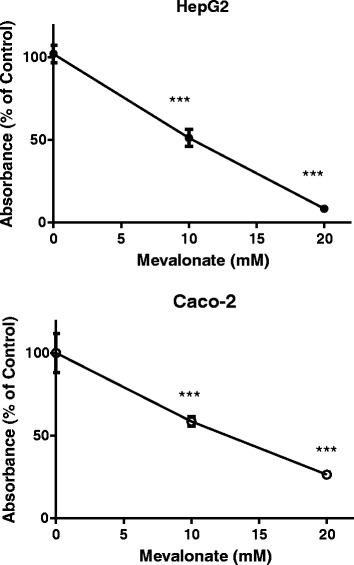
Cell proliferation after mevalonate treatment. Both HepG2 and Caco-2 cells were incubated with mevalonate for 48 h. The cell proliferation was determined by a kit from Trevigen, which was based on MTT reagent. Results are means ± SD from triple cultured wells in three separate experiments and expressed as percentage of the control. ***P < 0.005 compared with control

Since we found an increase of acid SMase in control cells with cell growth (Fig. 2), we further examined whether mevalonate treatment may affect cell proliferation. We treated both HepG2 and Caco-2 cells with mevalonate for 48 h, since at this time point, mevalonate was effective in inhibition of acid SMase in both cell lines (Fig. 1). We found that cell proliferation in HepG2 and Caco-2 cells was dose-dependently decreased. The inhibition is more potent for HepG2 cells than for Caco-2 cells (Fig. 5).

## Discussion

The interaction of cholesterol and SM has been an interesting research field for decades. Previous studies mainly focused on the effect of changed SM levels on cholesterol homeostasis, such as cholesterol trafficking and synthesis [1, 20, 22]. In the present study we address a question from the opposite direction, i.e. whether cholesterol synthesis influences SM levels *via* affecting SMase activity. The experiment was performed by treating the cells with mevalonate, the key molecule leading to cholesterol synthesis. We found that mevalonate at concentrations increased cholesterol levels, significantly inhibited acid SMase activity and increased intracellular SM levels in the cells. The effect was dose-dependent in both liver and intestinal cells. However the liver cells demonstrated higher sensitivity than intestinal cells, which supports the fact that mevalonate pathway is much more active in the liver than in the intestine [24], and liver has higher acid SMase activity than the intestine [16].

In general, increased SM levels can result from decreased degradation or increased synthesis. At least 3 types of enzymes can hydrolyze SM in human liver and intestine, i.e. the acid SMase, neutral SMases [13] and ENPP7 (also called alkaline SMase previously) [25]. In the present study, the overall activities of neutral SMases in the cell free lysates after mevalonate treatment for 24 and 48 h were not significantly changed, which, in our opinion, don’t supports a critical role of neutral SMases in the changed SM after mevalonate treatment. Due to the negative results, we did not further address a question whether different types of neutral SMase at different time points could be altered by mevalonate. In addition, ENPP7, although highly expressed in the intestine and human liver [26], is also unlikely responsible for the changes of SM induced by mevalonate, because in HepG2 cells ENPP7 is inactively mutant [27], and in Caco2 cells it is only expressed in polarized cells not monolayer cells [28]. The significant dose dependent and time-dependent reductions of acid SMase caused by mevalonate indicate that it is acid SMase that is responsible for the increased SM in the cells. Although SM levels can also be affected by de novo synthesis, previous studies have shown that the syntheses of SM and cholesterol are independent. Neither inhibiting cholesterol biosynthesis by statins nor enhancing cholesterol biosynthesis by mevalonate has effect on SM biosynthesis [29]. Cholesterol itself did not affect SPT activity [30].

The reduced acid SMase activity by mevalonate appears occurring mainly at post-translational levels. Although qPCR showed a mild reduction of acid SMase mRNA by 20 % after incubation with the highest concentration of mevalonate, the statistical significance was not reached. In addition, the Western blot did not reveal visible changes of acid SMase proteins. However, it might be worthwhile pointing out that Western blot showed an additional faint band below the major acid SMase band in all mevalonate treated samples but not in nontreated samples. This phenomenon may indicate a proteolytic action on acid SMase induced by mevalonate. It has been shown that post-translational modifications including glycosylation, phosphorylation, and proteolysis are important steps affecting acid SMase activity [14, 31]. In addition, mevalonate pathway generates both sterol and non-sterol products. The non-sterol products, which are mainly derivatives of isopenoids, have been shown to be able to interact with proteins [8, 9]. Of interest, we previously showed that triterpenoids such as ursolic acid and boswellic acid, which are natural sterols chemically derived from isopenoid, can affect acid SMase activity at post-translational levels [32, 33]. Further studies are necessary to characterize the underlying mechanisms leading to the decreased acid SMase activity triggered by mevalonate. In addition, whether the reduced acid SMase activity by mevalonate is a consequence of translocation of the enzyme is also a question requiring further investigation.

Many studies have shown that cells can regulate the content of SM and cholesterol in a delicate coordinate manner. Those studies mainly focus on the effects of changed cellular SM on cholesterol synthesis and trafficking [20, 29]. Our results cast a new insight that the coordinate regulation may occur also at the opposite direction, i.e. changed cholesterol synthesis also affects hydrolysis of SM through modifying acid SMase activity. The coordinate increase in cholesterol and SM may have clinical implications, as liver has important impact on the levels of both plasma LDL cholesterol and SM, both are risk factors in pathogenesis of atherosclerosis [10]. In addition, very recently, a cohort study shows that long term statin treatment increases risk of type-2 diabetes [34]. We previously showed that mevastatin could increase acid SMase in HepG2 cells [35]. The effect could increase the formation of ceramide that can induce insulin resistance through affecting protein kinase B pathway [36]. Our study thus may also have implications in pathogenesis of type-2 diabetes. Besides the changes of SM, we also found increased PC after mevalonate treatment. PC interacts with cholesterol similarly as SM but with lower affinity [37]. Increasing of PC by mevalonate may indicate the existence of a similar regulatory mechanism between cholesterol and PC. The mechanism underlying the increased PC levels after mevalonate treatment was not explored in the present study. Future studies should examine the changes of enzymes responsible for PC hydrolysis such as PC-specific phospholipase C, phospholipase D, and those involved in PC synthesis.

Finally we found that the activity of acid SMase was increasing with time in non-treated HepG2 cells and the increase was most obvious at the early stage of the cells after subculture, when the cells are rapidly proliferating. The results may indicate essential roles of acid SMase in cell proliferation at certain stages. In agreement with the hypothesis, mevalonate inhibited cell growth in both HepG2 and Caco-2 cells, with HepG2 cells being more sensitive than Caco-2 cells. We previously found that boswellic acid, a triterpenoid also inhibited Caco-2 cell proliferation associated with reduced acid SMase activity [32]. Since what the kit measured is the total number of living cells, the results did not provide information for the mechanism involved. Further comprehensive studies are required to characterize the underlying pathways related to cell proliferation and apoptosis. Of interest is the previous finding that statins, the drugs that inhibit HMGR, inhibited cell proliferation and induced apoptosis [38]. Future studies are required to clarify the discrepancy between the effects of statins and mevalonate on cell proliferation by detailed quantification of SM metabolites such as S1P, C1P and signals affected by statins. As pointed by others, some pleiotropic effects of statins may not be linked to its effects on cholesterol synthesis [38].

## Conclusion

The study reveals a novel regulatory mechanism between cholesterol synthesis and SM hydrolysis exerted by acid SMase. This type of regulation might be important in ensuring the coordinate changes of cholesterol and SM in the cells, and may have implications in cell survival, atherosclerosis and diabetes.

## Materials and methods

### Materials

HepG2 and Caco-2 cells were purchased from American Tissue Culture Collection. Mevalonate, antihuman acid SMase, anti β-actin, standard SM, PC, and cholesterol were purchased from Sigma Co (Stockholm, Sweden). Choline labeled SM (C^14^-SM) was prepared by Astra Zeneca (Lund, Sweden). ^3^H-labeled choline chloride was purchased from American Radiolabeled Chemicals Inc. (Malmö, Sweden). Primers for acid SMase and internal control gene (GAPDH) used in qPCR were synthesized by DNA Technology A/S (Risskov, Denmark) and the sequences have been described previously [32]. The MTT Cell Proliferation Assay (MTT-CPA) kit was purchased from Trevigen (Gaithersburg, USA). The Cholesterol Quantification kit and cDNA Synthesis kit for qPCR analysis were purchased from Cayman (Ann Arbor, USA) and Fermentas (Stockholm, Sweden), respectively.

### Cell Culture

HepG2 and Caco-2 cells were cultured in monolayer in RPMI-1640 medium and DMEM medium, respectively, containing 4.5 μg/l glucose, 2 mM glutamine, 10 % heat inactivated FBS, 100 IU/ml penicillin and 10 μg/ml streptomycin. Mevalonate was dissolved in the culture medium as a stock and added in the cell culture medium to final concentrations examined. In the control group, only the culture medium was added. After incubation, the cells were scraped, lysed and sonicated as described [32]. After centrifugation at 10000 g at 4 °C for 10 min, the activities of SMases in the homogenate were determined. For kinetic studies, one day after the subculture, the cells were treated with mevalonate for 8, 24, 48 and 72 h and the acid SMase activities were determined. The changes of the activity after mevalonate treatment were expressed as percentage of the values before mevalonate treatment (0 time value).

### SMase assay

The SMase activity was assayed as described before [39]. In brief, for acid SMase assay, 5 μl of sample was added in 95 μl 50 mM Tri-maleate buffer pH 5.0 containing 0.15 M NaCl, 0.12 % Triton ×100, and 80 pmol ^14^C-SM. After incubation at 37 °C for 30 min, the reaction was stopped by adding 0.4 ml of chloroform/methanol (2:1 v/v) followed by centrifugation shortly. After phase partition, the radioactivity in the upper phase containing cleaved phosphocholine was determined by liquid scintillation. The neutral SMase activity was assayed similarly but in 50 mM Tris–HCl buffer, containing 2 mM Mg^2+^, pH 7.4 [39, 40]. The activity was adjusted with the protein levels in the homogenate and expressed as percentage of the values in control group.

### Real time PCR for acid SMase mRNA and Western blot for acid SMase protein

The total RNA was extracted by TRIzol and cDNA was synthesized by a cDNA Synthesis kit for RT-PCR purchased from Fermentas. QPCR was performed in 20 μl volume containing 10 μl SYBR Green Supermix from Bio-Rad, cDNA samples, and primers on Bio-Rad iCycler system. The qPCR was programmed as 40 cycles of 95 °C for 15 s and 65 °C for 30 s. The melting curve was analyzed to confirm the specificity of the product amplified. The threshold (Cr) was analyzed by iQTM Optical System Software (Bio-Rad). The results were normalized with the levels of the housekeeping gene GAPDH. The Western blot was performed as described previously [32] . In brief, HepG2 cells were treated with mevalonate for 24 h and 50 mg proteins in cell lysate were subjected to 7.5 % SDS PAGE. The proteins were transferred to nitrocellulose membrane and blocked with 5 % non-fat dry milk. The membrane was then probed with antihuman acid SMase antibody (1:200) and then with second antibody (1:5000) conjugated with horseradish peroxidase. The specific acid SMase bands were identified by ECL reagents and visualized on Fuji Image. The membrane was then striped and re-probed with antibody against β-actin as a loading control.

### SM, PC and cholesterol assays

The changes of cellular levels of SM and PC were examined as previously reported [25]. In brief, the cells were first incubated with ^3^H-choline chloride (0.5 μCi/ml) to label the cellular SM and PC for 48 h and the excess of ^3^H-choline chloride was washed out thereafter. The cells were then incubated with mevalonate for 24 h. The total lipids in the homogenates of the cells were extracted according to Bligh and Dyer [41] and separated by TLC together with authentic standard SM and PC. The plate was developed by chloroform/methanol/ammonium hydroxide (65/25/4 v/v/v). The SM and PC bands were scraped according to the position of standards and the radio activities in SM and PC bands were measured by liquid scintillation. To examine the changes of cholesterol, the cells after mevalonate treatment were sonicated in isopropyl alcohol followed by centrifugation at 8000 g for 10 min. The cholesterol in the supernatant was assayed by a kit from Cayman according to the instructions of the company. The protein levels of the homogenates were determined and the levels of SM, PC and cholesterol were normalized with the protein contents in the samples and expressed as % of the controls.

### Cell proliferation assay

The cell proliferation was assay by MTT Cell Proliferation Assay (MTT-CPA) kit from Trevigen. The method is based on the ability of the cells to hydrolyze tetrazolium and generate purple formazan that can be quantitated spectroscopically at 595 nm on a microplate reader (Bio-Rad Model 680). The results reflect the total number of living cells. The determination procedure followed the instructions of the kit.

### Statistical analysis

The experiments were conducted in triple culture wells in 3 separate experiments. The results are presented as means ± SD, and analyzed by one way ANOVA followed by Dunnett’s test to compare the results in treated group with that in control group. *P <* 0.05 is considered to be statistically significant.

## Abbreviation

C1P: Ceramide-1-phosphate
HMGR: 3-hydroxy-3-methyl-glutaryl-CoA reductase
NPP7: Nucleotide pyrophosphatase phosphodiesterase 7
PC: Phosphatidylcholine
S1P: Sphingosine-1-phosphate
SM: Sphingomyelin
SMase: Sphingomyelinase
nSMase: Neutral SMase
SPT: Serine palmitoyltransferase
